# In-house genetic counseling increases the detection of abnormal karyotypes—a 26-year experience in prenatal diagnosis in a single tertiary referral hospital in Poland

**DOI:** 10.1007/s10815-020-01812-8

**Published:** 2020-05-19

**Authors:** Julia Bijok, Anna Kucińska–Chahwan, Diana Massalska, Alicja Ilnicka, Grzegorz Panek, Tomasz Roszkowski

**Affiliations:** 1 Department of Gynecologic Oncology and Obstetrics, Centre of Postgraduate Medical Education, ul. Czerniakowska 231, 00413 Warsaw, Poland; 2 Genetic Department, Institute of Psychiatry and Neurology, ul. Sobieskiego 9, 02957 Warsaw, Poland

**Keywords:** Changing patterns, Chromosomal anomalies, Genetic counseling, Genetic screening, Indications, Prenatal diagnosis

## Abstract

**Purpose:**

To evaluate the trends in prenatal diagnosis over 26 years in a tertiary referral hospital.

**Methods:**

A retrospective analysis of invasive prenatal procedures performed between 1991 and 2016. Maternal characteristics, indications for invasive diagnosis, and percentage of abnormal karyotypes were compared between periods according to guidelines implemented nationally and locally.

**Results:**

A total of 14,302 invasive prenatal procedures were performed. The proportion of invasive procedures performed for advanced maternal age, abnormal karyotype in a previous pregnancy, and maternal anxiety decreased from 71.1%, 17.8%, 8.9% in 1991 to 23.9%, 1.3%, and 2.3% in 2016 (OR 0.6, 0.8, and 0.9 for each 5 years, respectively; *p* < 0.001), while the proportion of invasive procedures performed for abnormal ultrasound increased from 2.2% in 1991 to 51.6% in 2016 (OR 1.9 for each 5 years; *p* < 0.001). Abnormal karyotype was found in 9.7%. The proportion of abnormal karyotypes increased significantly from 0.0% in 1991 to 15.7% in 2016 (OR 1.35 for each 5-year period; *p* < 0.001). The odds of abnormal karyotype increased after the implementation of the Ordinance of the Minister of Health in 2003 (OR 1.6), the National Prenatal Screening Program in 2007 (OR 2.2), and the in-house genetic counseling with combined first trimester screening in 2015 (OR 3.1).

**Conclusions:**

Significant changes in prenatal diagnosis led to a better selection of patients undergoing invasive prenatal procedures. The implementation of in-house genetic counseling was associated with an increased rate of the detection of abnormal karyotypes.

## Introduction

Chromosomal nondisjunction or breakage during gametogenesis or cell division may lead to chromosomal aberrations. Trisomy 21, which is the most common chromosomal abnormality at birth and the main cause of mental disability and congenital anomalies was first diagnosed postnatally in 1959 [1]. Invasive prenatal diagnosis was introduced in late 1960s [2]. Based on the fact that aging oocytes are at increased risk of aneuploidy, for many years, the most common indication for fetal karyotyping was advanced maternal age. However, age-based screening with a high false positive rate fails to diagnose a significant proportion of abnormal fetuses [3, 4]. Due to progress in ultrasound imaging as well as implementation of sensitive noninvasive biochemical markers of chromosomal aberrations, new trends in the prenatal diagnosis can be observed [5–9]. Moreover, incorporation of cell-free DNA testing (non-invasive prenatal testing (NIPT)) to routine practice has led to decline in invasive procedures observed in western countries [10–12]. Meanwhile, the recent meta-analysis of Akolekar et al. indicates a much smaller procedure-related risk of amniocentesis and chorionic villus sampling than previously reported [13], while advances in molecular genetic techniques enable a prenatal diagnosis of many syndromes that were previously beyond detection. All this put new light on invasive prenatal diagnosis.

As studies examining changing trends in prenatal diagnosis are scarce and usually based on data from western countries, we decided to evaluate the changes that took place in our referral center over 26 years of prenatal diagnosis and its impact on the percentage of abnormal karyotypes detected in our unit.

## Objectives

The aim of the study was to evaluate the changes in maternal and gestational age, the indications and type of invasive prenatal testing, and as well as its impact on the proportion of abnormal karyotypes diagnosed over a 26-year period in a tertiary referral prenatal center with regard to guidelines and changes implemented nationally and directly in our ultrasound unit.

## Material and methods

Prenatal care in Poland consists of public and private sector. Pregnant women are managed by specialists or trainees in Obstetrics and Gynecology. Patients at risk for fetal abnormality are counseled by clinical geneticists. In 2003, The Minister of Health issued an ordinance that recommended ultrasound examinations covered by the public health insurance system at 11–14, 21–26, and 33–37 weeks gestation. In 2007, National Prenatal Screening Program (NPSP) was introduced by the National Health Fund (NFZ) offering combined first trimester screening (cFTS) to women at high risk for fetal anomalies (age at delivery ≥ 35 years, family history of genetic or structural anomalies, or abnormal ultrasound findings in the current pregnancy). The main objective was to increase the availability of prenatal diagnosis; the implementation of biochemical screening, which before 2007 was available only in private out-patient clinics; and early identification of fetal anomalies. NIPT became clinically available in Poland in 2015 [14].

Our institution is a tertiary referral center for fetal medicine offering non-invasive prenatal diagnosis as well as invasive prenatal testing. The patients are referred for fetal evaluation from both public and private practices or from collaborating genetic departments for invasive procedures. Patients suspected of fetal anomalies are usually evaluated within 1 week from referral. Invasive procedures for fetal karyotyping at our institution are performed routinely from 1991. Since 2015, we offer genetic counseling directly in our unit by a geneticist–obstetrician experienced in prenatal ultrasound (AK-Ch).

We retrospectively examined invasive procedures performed in our Ultrasound Department between January 1, 1991 and December 31, 2016. Maternal age, gestational age at the time of invasive procedure, indications for prenatal testing, type of invasive procedure and fetal karyotype were analyzed. Procedures performed in twin pregnancies were excluded from analysis.

To simplify, the indications were divided into 7 subtypes. If there was more than one indication for invasive procedure for a particular patient, only one was analyzed using the following priority:

1. procedures performed for DNA analysis (i.e., increased risk for single gene disorders)
2. parental translocation carrier
3. abnormal ultrasound (fetal anomalies including isolated increased nuchal translucency (NT) ≥ 95th percentile) (AUS)
4. abnormal serum screening (ASS)
5. advanced maternal age (≥ 35 years) (AMA)
6. abnormal karyotype in a previous pregnancy, history
7. other indications, comprising mainly anxiety in case of parental distress in women under 35 years

All invasive procedures, namely amniocentesis (AC), chorionic villous sampling (CVS), and fetal blood sampling (FBS) were performed after informed consent by experienced operators. CVS at our institution is usually performed between 11 and 14 gestational weeks. AC is performed from 15 gestational weeks. Early AC (< 15 gestational weeks) were performed until 1998. Karyotyping was performed in the Genetic Department of the Institute of Psychiatry and Neurology in Warsaw after standard, flask culture of amniocytes, long-term culture of chorionic villi, or standard lymphocytes culture. Giemsa staining for G-banding (GTG) was used. From April 2013, rapid aneuploidy testing was performed on DNA extracted from uncultured amniotic fluid or chorionic villi as previously described using MLPA P095 probe kit (Multiplex ligation-dependent probe amplification) [15, 16].

Karyotypes 46,XX; 46,XY; and common polymorphisms were categorized as normal. Abnormal karyotypes comprised polyploidies, aneuploidies, structural aberrations, and complex chromosomal rearrangements.

We divided the study period into four periods (1991–2002, 2003–2006, 2007–2014, 2015–2016) according to the milestones that could be observed nationally and locally (the implementation of the Ordinance of the Minister of Health (OMH) in 2003, the introduction of NPSP in 2007, the implementation of in-house genetic counseling in 2015). Period of 2007–2016 was additionally subdivided into shorter periods to study the impact of in-house changes on the types of procedures and the rate of abnormal karyotype (i.e., the implementation of CVS for routine karyotyping in 2009, the introduction of rapid testing for common aneuploidies by MLPA in 2012).

Statistical analysis was performed using STATA 12 (StataCorp). Descriptive statistics was presented with means, medians, and proportions. Missing values were omitted. Multiple logistic regression was used to determine predictive factors of abnormal karyotype (maternal age, type of invasive procedure, gestational age, indications, sonographic abnormalities). *p* < 0.05 was considered statistically significant. A logistic regression model was used to determine the odds of abnormal karyotype after the implementation of the OMH in 2003, the introduction of the NPSP in 2007, and the implementation of in-house genetic counseling in 2015.

In case of descriptive, retrospective studies, institutional ethics committee permission is not necessary, nevertheless an internal bioethics committee approved the study design. All patients were informed and consented to use of their anonymized data for research purpose.

## Results

**Diagram 1:**
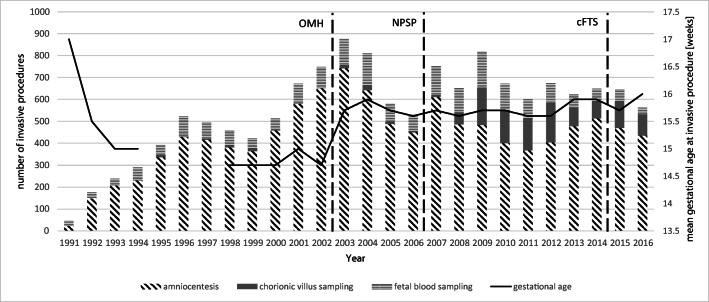
Invasive prenatal procedures performed yearly in the Department of Gynecologic Oncology and Obstetrics between 1991 and 2016. (OMH, the Ordinance of the Minister of Health; NPSP, National Prenatal Screening Program; cFTS, genetic counseling and combined first trimester screening in-house)

Over a 26-year period, a total of 14,302 invasive prenatal procedures was performed (AC 77.4%, *n* = 11,063; CVS 8.3%, *n* = 1190; FBS 14.3%, *n* = 2049). The number of invasive procedures per year is shown in Diagram 1. Maternal age, types, and indications for invasive procedures in different study periods are shown in Table 1.

**Table 1:** Maternal age, types, and indications for invasive procedures performed in the Department of Gynecologic Oncology and Obstetrics of the Postgraduate Center of Medical Education in Warsaw between 1991 and 2016 with the proportion of abnormal karyotypes before and after the implementation of the Ordinance of the Minister of Health in 2003, the National Prenatal Screening Program in 2007, and the in-house genetic counseling and combined first trimester screening in 2015

	1991–2002				2003–2006				2007–2014				2015–2016				*p*
Maternal age (years)	35.9				34.2				33.8				33.7				< 0.001
Invasive procedure																	< 0.001
AC	84.7%				83.0%				68.3%				74.5%				
FBS	14.3%				15.1%				15.5%				7.2%				
CV	1.0%				1.9%				16.2%				18.3%				
Indication	all	AC	FBS	CVS	all	AC	FBS	CVS	all	AC	FBS	CVS	all	AC	FBS	CVS	< 0.001
Parental	0.9%	1.0%	0.4%	0.0%	0.9%	1.1%	0.2%	0.0%	1.0%	1.2%	0.1%	0.8%	1.5%	1.7%	2.3%	0.5%	
DNA analysis	1.9%	1.3%	0.3%	81.3%	3.0%	1.7%	0.2%	81.5%	2.6%	2.0%	0.1%	7.6%	2.6%	1.9%	0.0%	6.4%	
AU	12.1%	4.9%	55.9%	4.2%	28.3%	17.7%	90.7%	0.0%	45.2%	31.2%	88.4%	62.9%	50.1%	38.6%	83.7%	83.6%	
ASS	1.2%	1.2%	1.4%	0.0%	1.3%	1.5%	0.5%	0.0%	1.1%	1.5%	0.1%	0.2%	15.8%	20.0%	3.5%	3.6%	
AMA	4.9%	77.3%	37.3%	8.3%	53.4%	63.1%	6.0%	3.7%	40.9%	53.1%	8.5%	20.5%	25.3%	32.3%	7.0%	4.1%	
History	71.0%	5.3%	2.3%	0.0%	3.5%	4.2%	0.0%	0.0%	2.7%	3.4%	0.1%	1.8%	1.9%	2.6%	0.0%	0.0%	
Other	8.0%	9.0%	2.4%	6.2%	9.6%	10.7%	2.4%	14.8%	6.5%	7.6%	2.7%	6.2%	2.8%	2.9%	3.5%	1.8%	
Abnormal karyotype	5.8%				9.1%				12.2%				15.9%				< 0.001

AC, amniocentesis; FBS, fetal blood sampling; CVS, chorionic villus sampling; AMA, advanced maternal age; ASS, abnormal serum screening; AU, abnormal ultrasound

The mean and median maternal age was 34.7 and 36.2 years, respectively (SD 5.9 years). A total of 54.9% patients (*n* = 7854) were > 35 years old. Maternal age decreased significantly over the study period from 35.7 years in 1991 to 31.0 years in 2016 (− 0.74 years for each 5-year period; *p* < 0.001). The mean gestational age at the invasive procedure was 17.0 weeks (CVS, 13.3 weeks; AC, 15.4 weeks; FBS, 23.9 weeks). The mean gestational age at the time of invasive procedure declined from 17.2 weeks in 1991 to 16.4 weeks in 2016 (− 0.18 week for each 5-year period; *p* < 0.001).

The most common indication for invasive testing was AMA accounting for 52.4% of all indications (*n* = 7490), followed by AU (*n* = 4421; 30.9%). History accounted for 3.5% procedures (*n* = 508), DNA analysis for 2.4% (*n* = 349), ASS for 2.4% (*n* = 341), and parental indications for 1.0% (*n* = 143). Other indications (i.e., maternal anxiety) comprised 7.4% (*n* = 1054).

Abnormal ultrasound comprised 77.5%, 61.4%, and 19.0% of indications in patients undergoing FBS, CVS, and AC, respectively (*p* < 0.05). In procedures performed for abnormal ultrasound, gestational age at invasive procedure decreased from 28 weeks in 1991 to 17 weeks in 2016 (− 2.4 weeks for each 5-year period; *p* < 0.001).

**Diagram 2:**
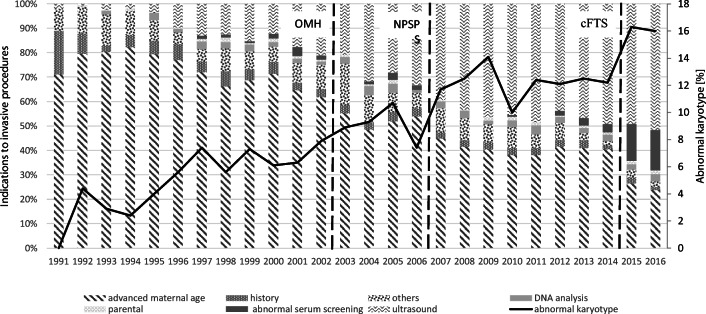
Indications for invasive prenatal procedures in the Department of Gynecologic Oncology and Obstetrics between 1991 and 2016. (OMH, the Ordinance of the Minister of Health; NPSP, National Prenatal Screening Program; cFTS, genetic counseling and combined first trimester screening in-house)

The proportion of invasive procedures performed for AMA, history, and maternal anxiety decreased throughout the study period from 71.1%, 17.8%, 8.9% in 1991 to 23.9%, 1.3%, and 2.3% in 2016 (OR 0.6, 0.8, and 0.9 for each 5 years; *p* < 0.001), respectively. Meanwhile, the proportion of invasive procedures performed for AU increased from 2.2% in 1991 to 51.6% in 2016 (OR 1.9 for each 5 years; *p* < 0.001). The proportion of parental indications remained stable throughout the study period at around 0.5–1.6% (n.s.). The proportion of procedures performed for DNA analysis increased slightly from 1.6% in 1993 to 3.0% in 2016, but the difference did not reach statistical significance (OR 1.1; *p* = 0.052) (see Diagram 2).

Abnormal karyotype was found in 9.7% of conclusive results (1345/13,862). The proportion of abnormal karyotypes increased significantly from 0.0% in 1991 and 4.4 % in 1992 to 15.7% in 2016 (OR 1.35 for each 5-year period; *p* < 0.001; see Diagram 2), and this increase was statistically significant both in women < 35 and ≥ 35 years old (*p* < 0.001). The odds of abnormal karyotype were increased after the implementation of OMH in 2003 (OR 1.6 95% CI 1.3–1.9) and the NPSP in 2007 (OR 2.2 95% CI 1.9–2.6) as well as the in-house genetic counseling (OR 3.1 95% CI 2.5–3.8).

CVS for routine karyotyping was introduced in 2009. Between 2009 and 2012, CVS comprised 23.2% of all invasive procedures compared with 15.5% in the years 2013–2016 (*p* < 0.001) after the implementation of rapid testing for aneuploidy.

There was a statistically significant difference in the proportion of invasive procedures performed for advanced maternal age and for abnormal serum screening between 2007 and 2014, when genetic counseling and combined first trimester screening were performed outside of our Ultrasound Department, and 2015 and 2016, when it started to be offered directly in our unit (40.8% vs 25.3%; 3.6% vs 15.7%; respectively, *p* < 0.001). The detection rate of abnormal karyotypes increased from 12.2% between 2007 and 2014 to 16.2% between 2015 and 2016 (*p* < 0.001).

## Discussion

We present a large data on prenatal diagnosis in the last three decades in a single tertiary referral center in Poland. Since our institution performed the largest number of invasive prenatal procedures in our country, it provides an illustration of trends in prenatal diagnosis observed in a middle European country over a long period of time. A major strength of our study apart from its duration and the size of its population is the fact that all procedures were performed or supervised by one experienced sonographer (T.R.), and the genetic testing was performed in a single genetic center.

Unlike other authors [8–11, 17–19], we did not observe a decline in invasive procedures neither after the introduction of NPSP with cFTS in 2007 or after the implementation of NIPT in 2013 [14], which suggests that the trends in our country do not reflect global changes in prenatal diagnosis. On the contrary, we observed a steady or even a slightly increased case load after 2007, which may be attributed to the increasing awareness of patients and doctors of the importance of prenatal diagnosis. There were many structural changes in Poland with opening of new fetal medicine units, changing accessibility to noninvasive testing as well as changes in the resources for disabled people provided in our country that could have had its contribution to the number of referred patients, but the analysis of such changes is beyond the scope of this paper. Noteworthy, Johnson et al. [11] in a tertiary referral center in Australia observed a relatively lower decline in invasive testing in comparison with national changes, which was explained by a high rate of procedures performed for abnormal ultrasound. This would be similar to our unit, where in the recent years abnormal ultrasound comprised over 50% of all indications. Similarly, Manegold–Brauer et al. reported a stable rate of invasive testing before and after the introduction of NIPT in a high-risk population [20]. However, as NIPT has a limited accessibility and its cost is not covered by the NFZ, we cannot draw any conclusions regarding its impact on the uptake of invasive prenatal testing.

The median maternal age at first pregnancy in Poland increased from 22 years in 1990 to 27 years in 2013, which is slightly lower than the median in EU-28 countries [21]. In the meantime, the maternal age in our study population decreased significantly over the study period. The mean gestational age at the invasive procedures decreased significantly over the years, which is a trend observed worldwide [17]. There was a significant decrease in gestational age in procedures performed for abnormal ultrasound, which could be attributed to the improvement in prenatal imaging as well as the introduction of national guidelines regarding mandatory first trimester anomaly scan at 11 to 13 + 6 gestational weeks according to the Fetal Medicine Foundation [22].

Similarly to the results of Valayatham et al. [23], advanced maternal age remained the main indication in our study group up until 2007, but the proportion of invasive procedures performed for AMA decreased over 3-fold throughout the study period. The most common indication afterwards was abnormal ultrasound which increased 25-fold over the years.

In 2015, we started to offer genetic counseling and combined first trimester screening in-house, and we observed an increase in the procedures performed for ASS from 3.4% in 2014 to over 15.1% in 2016 along with a decrease in procedures performed for advanced maternal age from 40.1% in 2014 to 26.5% in 2015. There was a significant increase in the rate of abnormal karyotypes (12% in 2014 vs 16% in 2015; *p* < 0.05)

The proportion of abnormal karyotypes in our study was almost 10% overall, which is similar to the results of Lichtenbelt et al. (9%) [5] but much higher than that reported by other authors (Konialis et al. 2.2% [15]; Xiao et al. 3.5% [24]) and reflects the high-risk population in our tertiary referral center. Meng et al. (9.6%) [9] reported a similar rate of genomic abnormalities in their study, but they used both routine karyotyping as well as aCGH for genetic testing. Similarly, Awomolo et al. in a recent study showed a high percentage of abnormal results, reaching 27% [19]. Nevertheless, the mode of testing was not stated in the paper and most likely comprised molecular genetics along with cytogenetics. In our unit, aCGH was introduced in 2017, and we achieved around 30% of abnormal results (unpublished data).

We observed a dramatic increase in the diagnostic yield over the study period, which was especially significant in the recent years (2015–2016). Noteworthy, this increase was statistically significant regardless of maternal age, which confirms a better selection for invasive procedures in all age groups. In 2016, chromosomal aberrations were found in almost 16% of tested fetuses, more than reported by Hui et al. and other authors [17]. We believe that this may be attributed to the fact that an uncommonly high rate of all invasive procedures performed in our center is due to abnormal ultrasound, since we offer an invasive procedure in case of any structural anomaly diagnosed in the fetus. Furthermore, before 2015, the patients were counseled by geneticists (or only obstetricians) and referred to our unit with suspicion of anomalies or increased risk for chromosomal anomalies. The quality of ultrasound and counseling varied in different centers. The in-house genetic counseling (by an obstetrician specialized both in fetal medicine as well as clinical genetics) that was implemented directly in our center in 2015 enabled us to offer first trimester combined screening (funded by the NFZ and not by the patient) and to counsel the patients referred for low-risk indications (such as maternal age or history) more conservatively, while offering invasive procedures to high-risk patients. The indications for invasive testing after 2015 comprised mostly abnormal ultrasound and abnormal serum screening, which are widely known to be high risk for abnormal karyotype.

An interesting local trend was a dramatic decrease in the proportion of CVS and FBS, which are associated with higher risk of pregnancy loss [25–27] after the introduction of rapid aneuploidy testing for common aneuploidies by MLPA in 2012 (from 24 to 17% and from 15 to 5%, respectively). Between 2009 and 2012, CVS comprised almost ¼ of all invasive procedures due to time gain in diagnosis. However, as amniotic fluid derives from different fetal tissues, it reflects the genuine fetal karyotype [28] and is preferred to chorionic villi or fetal blood for genetic testing. The indications for CVS in the recent years shifted almost completely to abnormal ultrasound in the first trimester and DNA analysis, which resulted in over 40% of chromosomal abnormalities diagnosed in chorionic villus samplings.

As expected, the implementation of national recommendations was significantly associated with an increase in the detection of abnormal karyotype in our study. This was most likely due to significant changes in the proportion of invasive procedures performed for abnormal ultrasound and abnormal serum screening that were associated with the implementation of new guidelines. The imaging technique has evolved tremendously owing to technical advances (high-resolution imaging) as well as a number of publications on the subject. Furthermore, patients’ awareness of the importance of prenatal diagnosis increased, while non-invasive screening has become widely accessible. One of the most interesting findings in our study was the fact that in-house genetic counseling by a geneticist–obstetrician experienced in prenatal ultrasound was associated with a significant increase in the detection rate of abnormal karyotypes, which in our opinion was mostly due to the fact that the baseline risk of patients undergoing invasive procedures changed during the study period owing to a better selection for testing. Further studies are needed to analyze the impact of different indications on the spectrum of chromosomal aberrations detected in invasive testing and will be presented in future.

## Conclusions

Over the years, significant changes took place in the field of prenatal diagnosis that led to a better selection of patients undergoing invasive prenatal procedures and an increase in the detection rate of chromosomal aberrations. Our data underlines the importance of combining perinatal medicine with clinical genetics and confirms the importance of genetic testing in case of fetal structural anomalies.

## Data Availability

Data available on request from the authors
